# Changes in Endogenous Carotenoids, Flavonoids, and Phenolics of Drought-Stressed Broccoli Seedlings After Ascorbic Acid Preconditioning

**DOI:** 10.3390/plants13243513

**Published:** 2024-12-16

**Authors:** Linqi Cai, Lord Abbey, Mason MacDonald

**Affiliations:** Faculty of Agriculture, Dalhousie University, Bible Hill, NS B2N 5E3, Canada; ln721551@dal.ca (L.C.); labbey@dal.ca (L.A.)

**Keywords:** antioxidant, ascorbate, *Brassica oleracea*, chlorophyll, photosynthesis, reactive oxygen species, water stress

## Abstract

Drought is an abiotic disturbance that reduces photosynthesis, plant growth, and crop yield. Ascorbic acid (AsA) was utilized as a seed preconditioning agent to assist broccoli (*Brassica oleracea* var. *italica*) in resisting drought. However, the precise mechanism by which AsA improves seedlings’ development remains unknown. One hypothesis is that AsA works via antioxidant mechanisms and reduces oxidative stress. This study aims to confirm the effect of varied concentrations of AsA (control, 0 ppm, 1 ppm, or 10 ppm) on seedling growth and changes in the antioxidant status of broccoli seedlings under regular watering or drought stress. AsA increased shoot dry mass, leaf area, net photosynthesis, and water use efficiency in watered and drought-stressed seedlings. AsA significantly (*p* < 0.001) increased carotenoid content in watered and drought-stressed seedlings by approximately 27% and 111%, respectively. Drought increased chlorophyll b, flavonoids, phenolics, ascorbate, and hydrogen peroxide production in control seedlings, but either had no effect or less effect on plants preconditioned with 10 ppm AsA. There was no improvement in reactive oxygen species scavenging in AsA-preconditioned seedlings compared to the control. The absence or reduction in biochemical indicators of stress suggests that preconditioned broccoli seedlings do not perceive stress the same as control seedlings. In conclusion, the consistent increase in carotenoid concentration suggests that carotenoids play some role in the preconditioning response, though the exact mechanism remains unknown.

## 1. Introduction

As the world’s population rises, the need for food and crops continually increases [1]. The global population is currently greater than eight billion and is projected to reach nine billion by 2050, with 8.9% of the world’s population still suffering from food insecurity [2]. Likewise, by 2050, the demand for food will be 35–56% more than in 2010 due to population growth [3]. Increased food demand due to a rising population and global climate change may lead to famine. Therefore, there is a need to increase agricultural productivity, efficiency, and technology to ensure a sufficient food supply for the growing world population [4].

In the current environment of global climate change, precipitation systems are shifting, resulting in an increase in aridity, a reduction in water quantity, and an increase in drought stress [4,5]. Approximately 26 percent of all environmental stresses is caused by drought; therefore, it is one of the most detrimental stresses for plants [4]. Drought stress has a detrimental impact on agricultural production by affecting the physio-biochemical characteristics of plants [6,7]. Thus, some strategies are needed to support plant growth under drought stress.

Broccoli is a popular agricultural crop that can offer great diversity and nutrition in human diets [8]. The global nutraceutical market is growing, especially in emerging countries, which provides an excellent opportunity for broccoli to be developed into value-added products [9]. Canada produced 40,010 t of fresh broccoli in 2021, up from approximately 36,340 t in 2020 [10]. However, broccoli yield can decline by 20–60% under drought stress, and only 10–15% of the broccoli’s entire aerial biomass is utilized, which is a severe problem in the existing agricultural production system [11]. Therefore, promoting broccoli production could be particularly beneficial in areas where food security is a concern and can help to improve the overall health and productivity of the plant, leading to increased aerial biomass [12].

One approach to improve plant tolerance to drought stress is to use seed preconditioning treatments that enhance the seedling’s ability to cope with stress conditions. Seed preconditioning means soaking seeds to confer certain benefits. Such benefits could include improved seedling vigor, germination rates, photosynthesis, stress tolerance, or yield [13,14]. A previous study found that preconditioning with ascorbic acid (AsA) increased photosynthesis and shoot dry mass by 83% and 52% [11]. Additionally, one study observed that AsA preconditioning increased wheat (*Triticum aestivum* L.) yields by about 26% [15]. What is more, seed preconditioning can increase seed vigor and protease activity in rice by 10% and 18%, respectively [14].

Preconditioning seeds with AsA is of particular interest, because it is affordable, readily available, and benefits several crops [11]. Ascorbic acid, also known as vitamin C, plays a vital role in plant growth and stress tolerance. It is involved in various metabolic processes, including photosynthesis, respiration, and antioxidant defense. Ascorbic acid has been reported to enhance plant growth and development and mitigate the negative effects of abiotic stress, such as drought [16]. A previous experiment showed that AsA preconditioning promotes growth and drought stress tolerance in broccoli seedlings [11]. It was observed AsA preconditioning increased the photosynthesis, root dry mass, shoot dry mass, water use efficiency, chlorophyll, relative water content, and leaf area of seedlings under drought stress. In addition, preconditioning with AsA reduced membrane damage in drought-affected seedlings [11].

The physiological mechanism through which AsA provides an advantage to broccoli remains unknown. The previous study offered three hypotheses [11]. First, since hydrated seedlings were larger and appeared more mature, they may have a greater chance of surviving drought. However, past studies showed that AsA promotes stress tolerance as early as germination, well before a plant could gain any additional benefit from increased growth [17]. Second, increasing the root: the shoot ratio may enable plants to obtain more water during drought. Nevertheless, according to the experiment design, all seedlings were grown in small containers where soil moisture did not differ amongst preconditioned treatments [11]. It seems implausible that increased root biomass was a key mechanism when root growth was restricted. Further, additional root biomass still does not explain the observations from previous studies [15,17], which observed improved stress tolerance as early as the germination stage. The third hypothesis is that AsA protects cellular membranes from stress-induced oxidative damage as an antioxidant. This hypothesis has never been tested and, therefore, is the basis for the proposed experiment.

This study aims to uncover whether AsA can improve growth and photosynthesis in broccoli through improvements in antioxidant status. The specific objectives are (1) to investigate the morpho-physiological responses, including the photosynthetic, chlorophyll, leaf area, and biomass indicators of broccoli seedlings with AsA preconditioning under drought stress and (2) to investigate changes in antioxidant compounds and reactive oxygen species (ROS) in AsA-preconditioned seedlings under drought stress.

## 2. Results

### 2.1. Morphological Response

**Figure 1:**
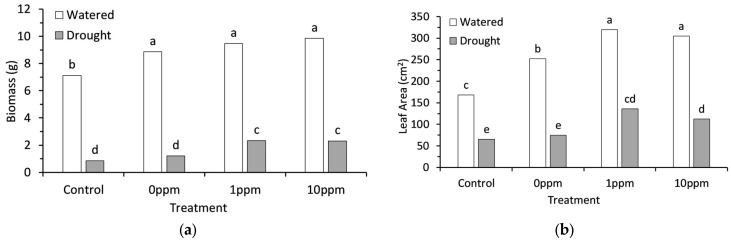
(**a**) Shoot dry biomass of broccoli seedlings; (**b**) leaf area of broccoli seedlings. Each figure compares four seed preconditioning treatments in both watered and drought conditions. Bars represent an average of 8 replicates for each treatment combination. Bars with different letters are significantly different based on Tukey’s multiple means comparison at 5% significance.

Regularly watered broccoli seedlings were seeds preconditioned with water (0 ppm), 1 ppm AsA, and 10 ppm AsA; they all had a significantly (*p* < 0.001) higher biomass than the watered control seeds that did not undergo preconditioning (Figure 1a). The regularly watered 10 ppm AsA treatment seedlings had the largest increase in biomass, which was 38% higher than the regularly watered control but was not significantly higher than other preconditioned treatments. Drought stress significantly (*p* < 0.001) decreased shoot dry mass in all treatments. However, the 1 ppm and 10 ppm AsA treatments were 170% and 92% higher than the control and 0 ppm AsA treatments during drought, respectively (Figure 1a).

Both drought stress and preconditioning treatments significantly (*p* < 0.001) influenced leaf area (Figure 1b). Drought stress decreased leaf area by an average of 64% across all preconditioning treatments, yet 1 ppm and 10 ppm AsA treatments consistently increased leaf area compared to their controls. Leaf area was increased by up to 90% in regularly watered seedlings and 106% in the drought treatment seedlings compared to their respective controls (Figure 1b).

### 2.2. Gas Exchange Response

**Figure 2:**
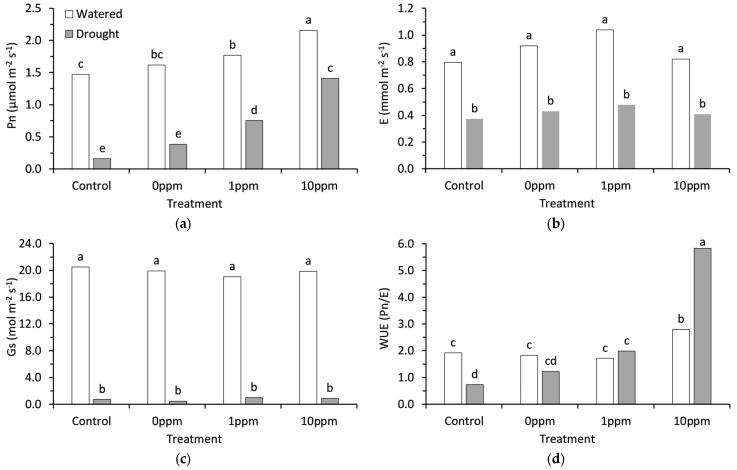
(**a**) Net photosynthesis (Pn); (**b**) evapotranspiration (E); (**c**) stomatal conductance (Gs); (**d**) water use efficiency (WUE) of broccoli seedlings. Each figure compares four seed preconditioning treatments in both watered and drought conditions. Bars represent an average of 8 replicates for each treatment combination. Bars with different letters are significantly different based on Tukey’s multiple means comparison at 5% significance.

Drought and seed preconditioning each had a significant (*p* < 0.001) effect on photosynthesis (Pn). The 10 ppm AsA treatment had the highest Pn in both watered and drought conditions. The 10 ppm AsA treatment increased Pn by 46% when watered and 780% under drought stress compared to their respective controls (Figure 2a). The photosynthetic activity declined to minimal levels in the drought-stressed control group. The photosynthetic activity in drought-stressed seedlings with 10 ppm AsA treatment were comparable to those of watered controls (Figure 2a).

In comparison to regularly watered group, transpiration (E) and stomatal conductance (Gs) were significantly (*p* < 0.001) reduced by 53% and 96%, respectively, in drought-stressed seedlings (Figure 2b,c). AsA preconditioning did not have a significant (*p* > 0.05) effect on transpiration (Figure 2b) or stomatal conductance (Figure 2c) under either watered or drought-stressed conditions. Compared to their respective controls, 10 ppm AsA preconditioning increased WUE from 0.732 to 5.828 under drought stress, representing nearly an 8-fold increase (Figure 2d). Similarly, under regular watering conditions, 10 ppm AsA preconditioning increased WUE from 1.913 to 2.799, which corresponds to a 1.5-fold increase (Figure 2d).

### 2.3. Biochemical Response

Neither drought stress nor seed preconditioning had any effect on Chl a content, but there was a significant (*p* < 0.001) interaction effect between stress and preconditioning. Regularly watered seedlings had an average Chl b concentration of 14.1 µg/g. Chl b increased by approximately 4-fold in the control and 2-fold in water-preconditioned seedlings during drought (Table 1). Seedlings with 10 ppm or 1 ppm AsA treatment had no significant change in Chl b under drought stress.

AsA preconditioning significantly (*p* < 0.001) increased carotenoid content in both watered and drought-stressed seedlings (Table 1). AsA treatment of 10 ppm increased carotenoid content by 27% in watered seedlings and 111% in drought-stressed seedlings compared to their respective controls. Under drought stress, the 10 ppm AsA treatment seedlings had a carotenoid concentration higher than regularly watered control seedlings. The 1 ppm AsA treatment was lower than 10 ppm treatment when watered or under drought stress but does not share a statistically difference (Table 1).

There was a significant (*p* < 0.001) interaction between drought stress and preconditioning for phenolics content. Drought stress increased total phenolic content approximately 2-fold in the control group, rising from 63.1 mg QE/g under watered conditions to 128.9 mg QE/g under drought conditions (Table 1). Among the drought treatments, preconditioning with 10 ppm AsA showed phenolic levels of 77.2 mg QE/g, which was comparable to the 0 ppm (65.3 mg QE/g) and 1 ppm (77.1 mg QE/g) AsA treatments but significantly lower than the drought-stressed control. Notably, in the watered condition, the 10 ppm AsA treatment exhibited the highest phenolic concentration at 72.8 mg QE/g, a 15% increase compared to the 0 ppm treatment and 33% higher than the watered control.

Drought stress significantly (*p* < 0.01) increased flavonoid content compared to watered seedlings, with the drought-stressed control exhibited a flavonoid concentration of 57.6 mg GAE/g, nearly double the 30.0 mg GAE/g observed in the watered control (Table 1). Flavonoid content was also significantly (*p* = 0.003) affected by AsA preconditioning. Under drought conditions, the 10 ppm AsA treatment exhibited a flavonoid concentration of 50.6 mg GAE/g, which was 11% higher than the 1 ppm treatment (45.5 mg GAE/g) and 26% higher than the 0 ppm treatment (40.3 mg GAE/g). However, this value was not significantly different from the drought-stressed control. In the watered group, the 10 ppm AsA treatment also showed higher flavonoid levels (39.8 mg GAE/g), representing a 33% increase over the watered control.

Drought stress significantly (*p* < 0.001) increased ascorbate content compared to watered seedlings. The ascorbate content has no significant (*p* > 0.05) difference across different AsA preconditioning treatments in regularly watered seedlings. The ascorbate concentration increased 7-fold in control seedlings due to drought but did not increase at all in the 0 ppm treatment. The ascorbate concentration increased by approximately 2.5- to 4-fold in the 1 and 10 ppm AsA treatments, though the concentration remained lower than that in the drought-stressed control seedlings (Table 1).

**Figure 3:**
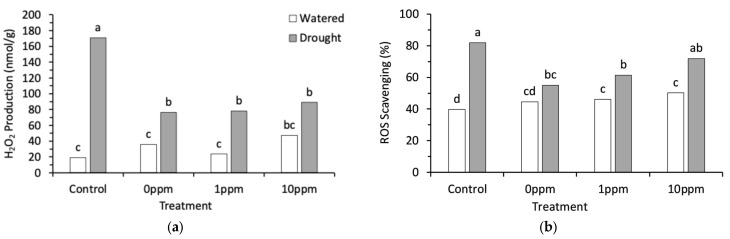
(**a**) Hydrogen peroxide production and (**b**) ROS scavenging in broccoli seedlings in 4 seed preconditioning treatments in broccoli seedlings that were watered or exposed to drought. Bars represent an average of 8 replicates for each treatment combination. Bars with different letters are significantly different based on Tukey’s multiple means comparison at 5% significance.

There was a significant interaction between stress and preconditioning treatments in H_2_O_2_ production and ROS scavenging (*p* = 0.002, *p* < 0.001, respectively). H_2_O_2_ production typically increased under drought stress. A 9-fold H_2_O_2_ production increase was observed in the drought-stressed control treatment seedlings. The effect of drought stress on H_2_O_2_ production in all preconditioning treatments increased by approximately 2- to 3-fold (Figure 3a). There was no significant (*p* > 0.05) increase in H_2_O_2_ production in the 10 ppm AsA treatments under drought stress (Figure 3a). ROS scavenging was significantly higher in watered 10 ppm AsA seedlings compared to the regularly watered control seedlings, but there was no significant (*p* > 0.05) difference in ROS scavenging between the 10 ppm AsA treatment and control under drought stress (Figure 3b).

### 2.4. Principal Component Analysis (PCA)

**Figure 4:**
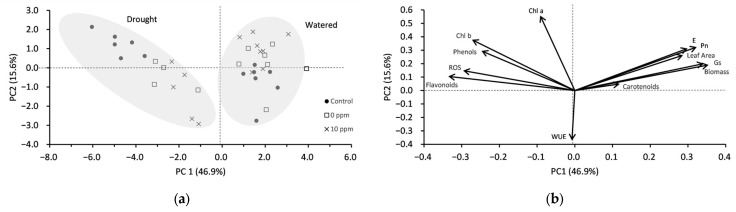
(**a**) Principal component score plot, with points identified by treatment. Shaded areas indicate clusters of points associated with drought or watered conditions; (**b**) principal component loading plot. Pn = net photosynthesis, E = transpiration, Gs = stomatal conductance, WUE = water use efficiency, Chl a and b = chlorophyll a and b, and ROS = reactive oxygen species.

The PCA score plot (Figure 4a) and loading plot (Figure 4b) provide insights into the variation and relationships among the growth and biochemical response variables. In the score plot (Figure 4a), data points from drought and watered treatments are clearly separated along the first principal component (PC1), which explains 46.9% of the total variance, indicating distinct differences between drought and watered responses. The second principal component (PC2) explains an additional 15.6% of the variance. The PCA loading plot (Figure 4b) reveals the contribution of multiple variables to these principal components. Plant growth parameters, such as Pn, E, Gs, leaf area, and biomass, are positively associated with PC1, while flavonoids and ROS are negatively associated with PC1. Conversely, responses such as Chl a and b show significant positive loadings on PC2, while WUE negatively influences PC2. These results indicate that plant growth parameters are the primary factors differentiating the samples along PC1; whereas, chlorophyll and WUE significantly influence the variance captured by PC2. This analysis underscores the importance of both growth and biochemical response in understanding the variability due to drought and seed.

## 3. Discussion

AsA increased Pn, WUE, leaf area, and shoot DM in watered and drought-stressed broccoli seedlings when compared to the control, which is consistent with previous results in tomato and broccoli [11,18]. AsA was not more effective than water preconditioning alone in watered seedlings, where that difference was significantly higher in previous research [11]. Perhaps the change in response could be attributed to plants being much larger in our study versus the previous study [11]. The relative response in leaf area, Pn, and WUE were much more consistent with previous work [11,18].

Pn appears to be a crucial response. Pn was significantly higher in AsA-preconditioned plants when watered and maintained at a high rate during drought. In fact, the 10 ppm AsA-preconditioned plants maintained Pn during drought at a rate comparable to the control plants that were watered, identical to previous results [11]. Pn also strongly influences the increases in other response variables, such as shoot DM, leaf area, and WUE. An increase in Pn improves the efficiency of CO_2_ fixation, which contributes to the production of carbohydrates essential for plant growth. This process allows seedlings to generate and allocate more resources toward building biomass, particularly in its shoots and leaves, thereby supporting overall development. In this study, an increase in Pn without a corresponding change in E enhanced WUE. E is important for nutrient uptake from the soil by maintaining water flow through the plant [19]. This occurs because broccoli seedlings may maintain E to prevent leaf overheating. In some drought scenarios, enough water might remain in deeper layers of soil, sustaining a steady level of E [20]. It may also be because broccoli seedlings fail to effectively convert or store the photosynthetic products, and nutrients accumulate without being efficiently used, reflecting an adaptation mechanism [19].

Chl a and b are the main photosynthetic pigments [21]. Chl a is the primary pigment to absorb light energy, primarily in the blue and red regions of the electromagnetic spectrum, thus driving the primary steps of photosynthesis [22]. In contrast, Chl b acts as an accessory pigment to supplement the light absorption spectrum by broadening the spectrum of absorbed light [23]. Drought stress had no significant effect on Chl a but increased Chl b in broccoli seedlings. This may be due to the differential responses of these Chl pigments to drought stress. Chl a content does not have a significant difference that was considered to be a mechanism to maintain minimal photosynthetic activity. Chl a plays a key role in transferring electrons during photosynthesis; it needs to be resilient to keep photosynthesis running smoothly, even under tough environmental conditions like drought [24]. The increasing Chl b content is thought to be a protective mechanism that helps maintain photosynthesis efficiency and prevent damage to the plant’s photosynthetic apparatus [25]. In addition, the increase in Chl b content helped the plant to capture more light and maximize its use of the limited resources available during a drought [26]. Plants treated with AsA did not accumulate additional Chl b during drought, which may be indicative that those plants were not experiencing stress at the same level as non-preconditioned plants. The response of Chl a and Chl b to drought stress are different when the duration of drought stress is different. One study found that Chl a and b contents in lettuce seedlings between control and drought-stressed group do not have a significant difference until day 6 of the treatment [27]. Another study observed that Chl a and b contents in cucumber plants between control and drought-stressed plants do not have a significant difference during the first three days, then decreased significantly by around 50% [28].

Flavonoids are a class of secondary metabolites produced by plants with a wide range of functions, including protection against biotic and abiotic stressors [29]. During drought stress, plants can experience oxidative stress, leading to the accumulation of ROS that can damage cellular structures and impair plant growth and development. Flavonoids have antioxidant properties and can help scavenge ROS, thus mitigating oxidative stress. In this study, flavonoid levels increased significantly in control seedlings under drought stress, reflecting their role as a protective response. However, AsA treatment seedlings did not show the same increase. This observation suggests that flavonoids may not be a primary component of the AsA seed preconditioning mechanism. Alternatively, the lack of obvious flavonoid accumulation could indicate that AsA treatment seedlings experienced lower oxidative stress under drought conditions, reducing the necessity for a flavonoid-mediated defense response. These findings align with other indicators, such as reduced H_2_O_2_ production in AsA treatment seedlings, further supporting the idea that AsA treatment lowers the oxidative stress under drought.

Phenolics are a diverse group of naturally occurring organic compounds characterized by the presence of multiple phenol units. Phenolics have antioxidant properties that involve in the plant’s defense mechanisms against drought stress by scavenging ROS [30]. This increased synthesis of phenolics under drought conditions helps mitigate cell damage from ROS, aid in maintaining cell structure and function [31]. However, like flavonoids, it seems that AsA does not function by altering phenolic content.

The total ascorbate content includes reduced ascorbate (AsA) and oxidized dehydroascorbate (DHA). AsA can act as an antioxidant and scavenge ROS, while DHA can be converted back to AsA by ascorbate reductase [32]. This conversion of DHA to AsA is a key component of the plant’s antioxidant defense system. This suggests that the increase in total ascorbate content results from increased biosynthesis and the recycling of ascorbate in response to drought stress.

H_2_O_2_ production is an estimate of oxidative stress in a plant [33]. H_2_O_2_ production increased in control plants due to drought but did not increase in plants preconditioned with AsA. This supports the idea that preconditioned plants are not experiencing the same level of stress as other plants. ROS scavenging activity refers to the ability of a plant cell to remove or neutralize ROS [34].

AsA preconditioning significantly increased carotenoid content in both watered and drought-stressed seedlings. Carotenoids play a dual role in protecting plants under stress. Carotenoids can dissipate excess energy and prevent the generation of ROS, thus protecting the photosynthetic machinery [35]. Additionally, carotenoids are potent antioxidants that that scavenge ROS and prevent oxidative damage to lipids, proteins, and other cellular components [36,37]. Additionally, drought stress can lead to the accumulation of abscisic acid (ABA) in plant tissues. ABA is a plant hormone that plays a key role in regulating plant responses to drought stress, like stomatal closure, to reduce water loss [38]. The increase in carotenoid content in broccoli seedlings during drought stress is related to ABA, because ABA can stimulate the biosynthesis of carotenoids in plant cells. ABA acts as a signaling molecule that can induce the expression of genes involved in carotenoid biosynthesis [39]. Therefore, another explanation is that the increase in carotenoid content in broccoli seedlings during drought stress is related to the role of ABA in regulating the biosynthesis of carotenoids. This increase in carotenoid content may also correlate with the improvement in WUE observed in AsA treatment seedlings. Carotenoids help maintain consistent photosynthetic activity under drought stress by protecting photosynthetic machinery and maintaining the integrity of chloroplasts. This sustained photosynthesis, combined with reduced transpiration due to ABA-mediated stomatal closure, contributes to higher WUE in AsA treatment seedlings. Specifically, the balance between maintaining photosynthesis and reducing water loss through transpiration suggests that carotenoids play a role in supporting efficient water use. Carotenoids may indirectly enhance the plant’s ability to optimize resource allocation under drought, thereby improving overall stress resilience.

The discovery of increased carotenoids may have significant implications for the agriculture industry. Carotenoids have antioxidant properties as well that can be associated with a reduced incidence of chronic diseases like cancer [40]. Beyond antioxidant properties, carotenoids have been linked to enhanced immune system, aiding in the function of both the innate and adaptive immune responses [41]. β-carotene has been shown to enhance the body’s ability to fight off infections by boosting the function of immune cells [42]. If the AsA pretreatment can be utilized on a larger scale, it could contribute to an increase in the production of broccoli rich in carotenoids, which could have significant health benefits for the general population. These results shed light on the complex interplay between plant metabolism and environmental stressors. AsA is an essential cofactor in many plants’ metabolic pathways, including photosynthesis. The increased carotenoid content observed in AsA-pretreated broccoli seedlings could be attributed to the upregulation of genes involved in carotenoid biosynthesis that are influenced by AsA levels [43].

In this study, the PCA analysis (Figure 4) provides insights into the relationships between plant physiological responses, biochemical parameters, and drought stress adaptation in broccoli seedlings. Positive correlations among Pn, WUE, biomass, and carotenoid content highlight the interdependence of these factors in contributing to the enhanced drought tolerance observed in AsA-preconditioned seedlings. These results align with previous findings that underscore the critical role of carotenoids in preserving photosynthetic efficiency and mitigating oxidative damage under stress conditions [41,42]. The PCA distinctly separates AsA-preconditioned seedlings from the control group, with Pn and WUE identified as key drivers of variability. This differentiation underscores the ability of AsA to modulate physiological traits critical for stress alleviation. Furthermore, the clustering patterns indicate reduced variability among AsA-treated seedlings, suggesting a more consistent response to drought stress compared to the untreated controls.

This study found AsA promotes growth and drought stress tolerance in broccoli seedling, but there is no evidence to support a direct role through antioxidant mechanisms. It appears carotenoids play some role in the effectiveness of AsA preconditioning, as carotenoids were the only class of compounds that increased due to AsA preconditioning compared to both the control and the water-preconditioned plants. However, there was no increase in ROS scavenging, so it is not likely that carotenoids are offering additional defense from antioxidant activity. Other responses indicative of stress, such as accumulation of Chl b, flavonoids, and ascorbate, did not occur in AsA-preconditioned plants. It is speculated that the increase in carotenoids may negate the perception of stress, though the exact mechanism through which this may be carried out remains unknown. The potential effects of AsA preconditioning on root architecture and microbial colonization should also be considered. Future studies can investigate these below-ground effects to further elucidate the role of AsA preconditioning in plant stress adaptation.

## 4. Materials and Methods

### 4.1. Experimental Design

This study was conducted in the Compost and Biostimulant Laboratory and greenhouse (45.3694° N, 63.2786° W) located in the Department of Plant, Food, and Environmental Sciences, Dalhousie University, Faculty of Agriculture, Truro, between November 2022 and December 2022. This experiment has two variables of interest and one blocking factor. The first variable of interest was seed preconditioning, which had four levels: no preconditioning (control), preconditioning with water (0 ppm), preconditioning with 1 ppm AsA, and preconditioning with 10 ppm AsA. The second variable was the two-tiered stress level, where plants were watered or exposed to drought. All broccoli seedlings were grown and watered for four weeks. Then, half of the seedlings were deprived of water for one week to impose drought, while the remaining seedlings continued to receive water. The blocking factor was determined by placing seedlings in one of three distinct locations within the greenhouse. The experiment was replicated three times, requiring a total of 72 broccoli seedlings in total. Each seedling represented an experimental unit.

### 4.2. Seed Preconditioning

A 1000 ppm stock solution of AsA (Sigma-Aldrich, Oakville, ON, Canada) was prepared in deionized water. The stock solution was diluted to 10 ppm and 1 ppm; whereas, the 0 ppm AsA treatment consisted of deionized water alone. The three preconditioning treatments were each put in a 250 mL flask. Each flask was filled with 25 seeds and placed on a rotary shaker at 150 rpm, as described in a previous study [18]. After 24 h, the contents of the flask were passed through a sieve, and the seeds were dabbed dry. The seeds of the control group were also placed in a flask and onto the rotary shaker but otherwise left untreated.

### 4.3. Growing Conditions

Broccoli seeds were sown into 10 cm pots utilizing Promix (Halifax Seed, Halifax, NS, Canada) as substrate. Promix is a growing medium commonly used in horticulture and research applications. Promix is composed primarily of Sphagnum peat moss, which makes up 75–85% of the blend [44]. Promix also includes perlite (10–20%) and vermiculite (5–10%), which work together to enhance drainage and improve root oxygenation [44]. The pH of Promix is adjusted to 5.5–6.5 using dolomitic and calcitic limestone, which provides a stable pH range ideal for nutrient uptake [44]. Additionally, the substrate contains a wetting agent that facilitates uniform water distribution throughout the growing medium. Within the greenhouse, three trays in various locations acted as experimental blocks. Six pots of each preconditioning treatment were allocated randomly to each tray, for a total of 24 pots per tray. Pot arrangement within each block was randomized using Minitab 19.0 software (Minitab Inc., State College, PA, USA).

All seedlings were cultivated under identical circumstances for three weeks. Temperatures in the greenhouse were adjusted to 20 °C by day and 15 °C at night. For the first four weeks, all seedlings were given 200 ppm of 20-20-20 fertilizer, 5 ppm of iron chelate, and 2 ppm of micro-chelate (all from Halifax Seed, Halifax, NS, Canada). However, half of the samples were deprived of water and fertilizer for one week after the four-week cultivation to induce drought stress. Soil moisture was measured throughout the experiment; otherwise, all other response variables were measured at the conclusion of the experiment.

### 4.4. Growth, Stress, and Gas Exchange

#### 4.4.1. Gas Exchange

Pn, E, and Gs were monitored with an LCA-4 Gas Exchange System (ADC Bioscientific, Hoddesdon, UK) based on the method described by a previous study [11]. The gas exchange chamber was clamped onto the most recent fully expanded leaf and allowed to equilibrate. Values were measured at 30 s, 60 s, and 90 s. Each of Pn, E, and Gs was reported as the mean of these three measurements. WUE was determined as the ratio of Pn/E.

#### 4.4.2. Leaf Area

ImageJ processing and analysis software (https://imagej.net/ij/, National Institutes of Health, Bethesda, MA, USA) was used to assess leaf area. At the soil level, a scaled white paper backboard was put around the seedling stems, and photographs were shot from above at a height that allowed the backboard to completely fill the frame. The backboard included a reference ruler to measure distance. The ruler was used to set the scale in each image. Then, pictures were converted into binary images, and leaves were selected with the flood fill tool to determine the full canopy area.

#### 4.4.3. Biomass

The whole seedling was extracted from the pot, separating it into shoot and root components. Any adhering soil of the shoots was cleaned. Subject to a drying protocol, the shoots were dried at 80 °C for 48 h in hot air to ensure complete moisture removal. Following the drying period, the shoot’s dry mass was determined through weighing.

### 4.5. Biochemical Analysis

#### 4.5.1. Chlorophyll and Carotenoids

The levels of total Chl a, Chl b, and carotenoids were determined according to the method described by [45] with little modification. To start, 0.2 g of the ground leaf sample was transferred to a sterile 50 mL falcon tube, and then, 10 mL of acetone (80% *v/v*) was added. The mixture was vortexed for 1 min and then centrifuged for 15 min at 12,000× *g.* Then, 1 mL of supernatant was transferred to a cuvette. Using 80% acetone as a blank, absorbance was measured using a UV-Vis spectrophotometer (Tecan Infinite^®^ M200 PRO, Morrisville, NC, USA) at 646.8 and 663.2 nm. The concentration of Chl a and b were calculated using Equations (1) and (2) below. In addition, the absorbance of the carotenoid was measured at 470 nm using Equation (3).

(1) $$Chl\;a\left(\frac{\mu g}{g}\right)=12.25A_{663.2}-2.79A_{646.8}$$

(2) $$Chl\;b\left(\frac{\mu g}{g}\right)=21.5A_{663.2}-5.1A_{646.8}$$

(3) $$Carotenoid\left(\frac{\mu g}{g}\right)=\frac{\left(1000A_{470}-1.8Chl\;a-85.02Chl\;b\right)}{198}$$

#### 4.5.2. Total Flavonoids

Total flavonoids were determined using a modified procedure [46]. An amount of 0.2 g of ground leaf sample was homogenized with 2.5 mL of 95% (*v/v*) methanol. The mixture was centrifuged for 10 min at 13,000× *g*, and 500 uL of the supernatant was transferred to a fresh tube. Each tube received 1.5 mL of 95% (*v/v*) methanol, 0.1 mL of 10% (m/v) AlCl_3_, 0.1 mL of 1 M potassium acetate, and 2.8 mL of distilled water. After vortexing and incubating the mixture for 30 min at room temperature, the absorbance was measured at 415 nm against a blank. Using a quercetin standard curve, the flavonoid content was calculated using Equation (4).

(4) $$Flavonoid\left(\%\right)=Concentration\left(\frac{\mu g}{mL}\right)\times \frac{volume\;of\;extract\;(mL)}{mass\;of\;extract\;(\mu g)}\times 100$$

#### 4.5.3. Total Phenolics

The total phenolic content was measured according to the method of Folin–Ciocalteu assay with little modification [47]. A 0.2 g ground leaf sample was homogenized in 2 mL of ice-cold 95% (*v/v*) methanol. Then, the sample was kept at room temperature in darkness for 48 h. The mixture was centrifuged at 13,000× *g* for 5 min, after which 100 uL of the supernatant was transferred to a fresh microfuge tube, and 200 uL of 10% Folin–Ciocalteu reagent was added. The mixture was vortex for 5 min. Then, 800 uL of 700 nM Na_2_CO_3_ was added, vortexed for 1 min, and incubated at 25 °C for 2 h. The concentration was determined by measuring absorbance at 765 nm with a UV-vis spectrophotometer. The total phenolic content was represented as mg gallic acid equivalents per gram of sample.

#### 4.5.4. H_2_O_2_ Production

H_2_O_2_ production was determined using a procedure from [48]. A 0.2 g ground leaf sample was homogenized in 1 mL of 0.1% (*w/v*) trichloroacetic acid (TCA). The mixture was centrifuged at 16,000× *g* for 10 min at 4 °C. A 200 µL aliquot of supernatant was mixed with 200 µL of 100 mM potassium phosphate buffer (pH 7.0) and 800 µL of 1 mM potassium iodide. The reaction was developed for 1 h in darkness and the absorbance measured at 390 nm against a blank that consisted of 0.1% TCA in the absence of leaf extract. H_2_O_2_ production was calculated from a pre-determined standard curve.

#### 4.5.5. ROS Scavenging–DPPH (2,2-Diphenyl-1-picrylhydrazyl) Assay

A 0.2 g ground leaf sample was placed in a 15 mL tube with 15 mL of pure methanol. The mixture was agitated and centrifuged at 10,000× *g* for 10 min. The supernatant was transferred to a new tube, and then, 10 mL of methanol was added to the original tissue pellet for a second extraction. The supernatant from both extractions was combined and made to a volume of 25 mL using pure methanol. A 100 uL aliquot of the supernatant was transferred to a separate tube, followed by the addition of 2.9 mL of 60 M DPPH methanolic solution. Immediately, the reaction mixture was incubated in darkness at 25 °C for 1 h. A methanol blank was used to measure the absorbance of the reaction mixture at 515 nm. The ROS scavenging was calculated using Equation (5), where A_b_ represents the absorption of the blank sample, and A_s_ is the absorbance of the test sample.

(5) $$ROS\;scavenging\left(\%\right)=\frac{A_{b}-A_{s}}{A_{b}}\times 100$$

#### 4.5.6. Total Ascorbate Content

Approximately 0.2 g ground leaf sample was homogenized in 1.5 mL of ice-cold 5% TCA. The mixture was vortexed for 2 min and centrifuged for 10 min at 12,000× *g* and 4 °C. Then, 100 µL of the supernatant was transferred to a separate tube, followed by the addition of 400 µL of phosphate buffer (150 mM potassium dihydrogen phosphate (KH_2_PO_4_ at pH 7.4), 5 mM ethylenediaminetetraacetic acid (EDTA), and 100 uL of 10 mM dithiothreitol (DTT). A solution of 400 uL of 10% (w/v) TCA, 400 uL of 44% (w/v) orthophosphoric acid, 400 µL of 4% (w/v) α- α-1-dipyridyl in 70% (*v/v*) ethanol, and 200 µL of 30 g/L ferric chloride (FeCl_3_) was added to obtain color. The absorbance was measured at 525 nm after 60 min of incubation at 40 °C in a shaking incubator. The total ascorbate content was calculated using the conventional L-ascorbic acid curve and expressed in mol/g.

### 4.6. Statistical Analysis

Minitab was used to conduct an analysis of variance (ANOVA) on the data (ver. 19.0, Minitab Inc., State College, PA, USA). Variables included in the model were preconditioning treatment, stress, preconditioning by stress interaction, and the blocking factor. Statistical assumptions of normality, homogeneity, and independence were met without the need for transformation. Means were separated using Tukey’s post hoc test when the ANOVA detected differences at 5% significance.

## Figures and Tables

**Table 1:** Concentration of chlorophyll a, chlorophyll b, carotenoids, flavonoids, phenolics, and ascorbate in 4 seed preconditioning treatments in broccoli seedlings that were watered or exposed to drought. Means with different letters within a column denote a significant difference as determined by Tukey’s multiple mean comparison at 5% significance. Columns without letter groupings did not have significant differences between treatments.

Status	Treatment	Chl a (µg/g)	Chl b (µg/g)		Carotenoids (ug/g)		Phenolics (mg QE/g)		Flavonoids (mg GAE/g)		Ascorbate (umol/g)	
Watered	Control	24.6	11.3	c	6.7	b	63.1	b	30.0	c	0.2	c
	0 ppm	26.4	12.9	c	7.4	b	71.0	b	30.8	c	0.3	c
	1 ppm	27.1	14.9	c	7.6	ab	64.4	b	28.9	c	0.2	c
	10 ppm	31.7	17.3	c	8.5	a	72.8	b	39.8	b	0.3	c
Drought	Control	32.4	43.0	a	3.8	c	128.9	a	57.6	a	1.4	a
	0 ppm	31.3	27.4	b	6.4	b	65.3	b	40.3	b	0.5	bc
	1 ppm	24.3	15.3	c	8.5	a	77.1	b	45.5	b	0.8	b
	10 ppm	28.3	18.1	c	8.0	a	77.2	b	50.6	ab	0.8	b

## Data Availability

The raw data supporting the conclusions of this article will be made available by the authors on request.
